# Protective Effect of *Brassica rapa* Polysaccharide against Acute High-Altitude Hypoxia-Induced Brain Injury and Its Metabolomics

**DOI:** 10.1155/2022/3063899

**Published:** 2022-04-23

**Authors:** Xuemei Zou, Hailing Yang, Qiuyue Li, Ning Li, Ya Hou, Xiaobo Wang, Xianli Meng, Jia Yu, Yi Zhang, Ce Tang, Tingting Kuang

**Affiliations:** ^1^State Key Laboratory of Southwestern Chinese Medicine Resources, School of Ethnic Medicine, Chengdu University of Traditional Chinese Medicine, Chengdu 611137, China; ^2^Innovative Institute of Chinese Medicine and Pharmacy, Chengdu University of Traditional Chinese Medicine, Chengdu 611137, China; ^3^Pharmacy Intravenous Admixture Services, The Affiliated Traditional Chinese Medicine Hospital of Southwest Medical University, Luzhou 646600, China; ^4^School of Pharmacy, Chengdu University of Traditional Chinese Medicine, Chengdu 611137, China

## Abstract

*Brassica rapa* L., a traditional Tibetan medicine, has been wildly used for treating plateau disease. Polysaccharide is an important chemical component in *B. rapa*. The present study aimed to evaluate the effect of *B. rapa* polysaccharide (BRP) against acute high-altitude hypoxia (AHH) induced brain injury and its metabolic mechanism. The rats were randomly divided into six groups: control group, AHH group, Hongjingtian oral liquid group, and three BRP groups (38, 75, and 150 mg/kg/d). Serum levels of superoxide dismutase (SOD), malondialdehyde (MDA), glutathione (GSH), oxidized glutathione (GSSG), and lactate dehydrogenase (LDH) were detected by commercial biochemical kits. Hippocampus and cortex histopathological changes were observed by H&E staining and Nissl staining. Neuronal apoptosis was observed by TUNEL staining. The protein and gene expression of Caspase-3, Bax, Bcl-2, p-PI3K, PI3K, p-Akt, Akt, HIF-1*α*, microRNA 210, ISCU1/2, and COX10 were detected by western blotting and qRT-PCR. Then, a brain metabolomics method based on UPLC-Q-Exactive-MS was performed to discover potential biomarkers and analyze metabolic pathways. It was found that BRP decreased levels of MDA, LDH, and GSSG, increased GSH and SOD, reduced the pathological changes, inhibited apoptosis, and activated the PI3K/Akt/HIF-1*α* signaling pathway as evidenced by increased phosphorylation of PI3K and Akt, enhanced protein expression of HIF-1*α* and gene levels of microRNA210, ISCU1/2, and COX10. Furthermore, 15 endogenous potential biomarkers were identified in the brain through metabolomics analysis. BRP can regulate 7 potential biomarkers and the corresponding metabolic pathways were mainly associated with pyruvate metabolism and glycolysis/gluconeogenesis. Collectively, BRP has a clear protective effect on AHH-induced brain injury and its mechanisms may be related to ameliorate oxidative stress injury, inhibit apoptosis by activating PI3K/Akt/HIF-1*α* signaling pathway, and reverse metabolic pathway disturbances.

## 1. Introduction

In the plateau environment, the atmospheric pressure drops and the oxygen content is low, which causes the decrease of oxygen partial pressure and blood oxygen saturation. Acute high-altitude hypoxia (AHH) is defined as a disease of the body's incomplete ability to adapt to the low-pressure and hypoxic environment when entering the plateau area from low altitude [1]. This hypobaric hypoxia triggers a series of compensatory changes in organs, tissues, and cells, such as acute mountain sickness, high-altitude cerebral edema, and high-altitude pulmonary edema [2–4]. Symptoms include deepening and accelerated breathing, accelerated heart rhythm, headache, fatigue, insomnia, dizziness, nausea, and vomiting, which can be life-threatening in severe cases [5–7]. Brains, especially the hippocampus, corpus striatum, and cerebellum, are sensitive and vulnerable to hypoxia [8]. The pathological features of hypobaric hypoxia-induced brain injury include morphological changes, mitochondrial dysfunction, and upregulation of proteins and genes associated with apoptosis [9]. Therefore, how to improve AHH-induced brain injury has garnered widespread attention recently. The phosphoinositide 3-kinase (PI3K) and the downstream effector protein kinase B (Akt) belong to a conserved family of signal transduction enzymes, which are involved in regulating glucose metabolism, oxidative stress, and apoptosis [10–12]. Hypoxia inducible factor-1 alpha (HIF-1*α*) is subjected to regulation by the PI3K/Akt [13–15]. It is one of the most crucial signaling molecules which mediates the responses of mammalian cells to hypoxia by inducing the expression of adaptive gene products [16]. Previous studies have shown overexpression of microRNA210 induced by upregulation of HIF-1*α* was found to decrease expression of iron-sulfur cluster scaffold (ISCU1/2) and cytochrome c oxidase assembly protein (COX10) under hypoxic challenge [17, 18].

The drugs currently used to prevent high-altitude sickness include acetazolamide, dexamethasone, montelukast, and aspirin, but these drugs have varying degrees of toxic side effects [19]. Thus, researchers' attention has been transformed into natural medicine. Tibetan medicine has obvious advantages in the prevention and treatment of plateau diseases because of its unique plateau geographical background. Tibetan medicine “Hongjingtian” (Radix et *Rhodiola crenulata*) and its preparations are recognized as good anti-hypoxia drugs with few toxic side effects [20, 21]. Although “Hongjingtian” has remarkable effect, its development and utilization are limited due to the sharp reduction of resources caused by poor growth environment and immature artificial cultivation.

*Brassica rapa* L. has a long history of dual use of medicine and food in Tibetan folk, which is widely planted in Tibetan areas and is rich in resources. Its root has been recorded as Brassicae Radix, a traditional Tibetan medicine also used to prevent high-altitude hypoxia, which is called MANJING or YUANJINN [22]. According to the records in famous classic works of traditional Tibetan medicine “*Four-Volume Medical Code*” and “*Jing Zhu Materia Medica*,” MANJING has the effects of nourishing, detoxification, helping digestion, and can prevent and treat plateau disease [23]. In recent years, polysaccharides isolated from natural plants become a focus in drug development because of their wide range of sources and safety. Polysaccharides are also the main component of *B. rapa.* Our previous studies found that *B. rapa* polysaccharide (BRP) has the functions of anti-hypoxia, anti-apoptosis, and improving immunity [24–26], but the function and mechanisms of BRP against AHH-induced injury remain unclear.

Metabonomics is an established but still expanding field of research in terms of seeking differences between the metabolic profiles of the test and control groups [27]. It is found that high-altitude hypoxia will lead to the lack of active oxygen in the body, and the corresponding metabolites will change accordingly [28]. Relying on metabonomic methods to analyze the changes of related endogenous metabolites can further reveal the occurrence and mechanism of AHH and provide a basis for clinical diagnosis. Nuclear magnetic resonance (NMR) and ultra performance liquid chromatography mass spectrometry (UPLC-MS) are the main methods of metabonomics. The latter has better reproducibility and detection limits, and increased chromatographic resolution, which can assess all metabolites in biological samples [29].

Thus, in this study, the AHH rat model was used to evaluate the therapeutic effect of BRP under hypobaric hypoxia. The levels of oxidative stress indexes, apoptotic cytokine, and PI3K/Akt/HIF-1*α* signaling pathways as well as the activities of key enzymes were determined. And based on UPLC-Q-Exactive-MS technology, the metabolomics method was applied to identify potential biomarkers and related metabolic pathways in brain tissue, which would be a valuable reference for further study and clinical application of BRP against AHH-induced brain injury.

## 2. Materials and Methods

### 2.1. Plant Material

*B. rapa* was collected from Maerkang County (Sichuan, China) and authenticated by Prof. Zhang Yi. The voucher specimens (MJ11-MEK-2) of the plant material are reserved in the herbarium of the College of Ethnic Medicine, Chengdu University of Traditional Chinese Medicine.

### 2.2. Preparation of BRP

The method of preparing BRP is the same as our previous research. Whole roots of *B. rapa* were milled into a coarse powder. The powder was refluxed with 80% ethanol for 3 h and extracted twice. Then, the filtered residues were refluxed with water for 2 h and extracted thrice. All filtrates were concentrated and mixed with 95% ethanol to 80% ethanol at 4°C overnight. The crude BRP was obtained by centrifugation and further deproteinated with chloroform-n-butanol (5 : 1). Then, the aqueous fraction was dialyzed with water and precipitated again by adding ethanol. After centrifugation, the precipitate was dissolved in water and then lyophilized to obtain BRP. The main components of BRP are galactose, rhamnose, galacturonic acid, and anhydrous glucose [25].

### 2.3. Chemicals and Reagents

Hematoxylin (20170308) was obtained from Thermo Fisher Scientific, and PAS staining fluid (20170823) was purchased from Solarbio Science & Technology Co., Ltd (Beijing, China). 4% paraformaldehyde (20170515) was provided by Cologne Chemicals Co., Ltd (Chengdu, China). PBS buffer reagent (13C01A30) was obtained from BOSTER Biological Technology Co., Ltd (Wuhan, China). Commercial kits were used to measure the activities of SOD (A001-3), GSH (A006-2), GSSG (A061-1), MDA (A003-1), and LDH (A020-2) which were purchased from Nanjing Jiancheng Bioengineering Institute (Jiangsu, China). BCA assay kit (AR0146), Hypersensitive ECL chemiluminescence kit (4AW011-100), and TUREscript 1st Stand cDNA SYNTHESIS kit(KL101-252)were, respectively, obtained from Boster Biological Technology Co., Ltd (Wuhan, China), Beijing 4A Biotech Co., Ltd (Beijing, China), and aidlab Biotechnologies Co., Ltd (Beijing, China). Antibodies of Bcl-2 (ab196495), Bax (ab32503), Caspase-3 (ab13847), *β*-actin (ab8227), HIF-1*α* (PB0245), p-PI3K (AF3241), PI3K (AF6241), p-Akt (#4060 s), and Akt (#4691 s) were, respectively, obtained from Abcam (Shanghai, China), Boster Biological Technology Co., Ltd (Wuhan, China), Affinity Biosciences Co., Ltd (Jiangsu, China), Cell Signaling Technology. Methanol (HPLC grade), formic acid (HPLC grade), and 2-chlorophenylalanine were purchased from Aladdin Bio-Chem Technology Co., Ltd (Shanghai, China); acetonitrile (HPLC grade) was bought from Wokai Biotechnology Co., Ltd (Beijing, China).

### 2.4. Animals and Treatment

Adult male SD rats, aged 2-3 months and weighing 210 ± 10 g, were purchased from Sichuan Dashuo Experimental Animal Co., Ltd (Chengdu, China; the animal license permit number license number: SCXK (Chuan) 2015-030). The rats were kept in the animal observation room of Chengdu University of Traditional Chinese Medicine under constant conditions (20-25°C, 12 h dark/light cycle, 40%-60% relative humidity). The study protocol involving animals was conducted in accordance with National Institutes of Health Guide for the Care and Use of Laboratory Animals and with the approval of the ethical committee of Chengdu University of Traditional Chinese Medicine, Chengdu, China.

After 7 days of acclimatization, the rats were randomly divided into six groups (six rats per group): normal control group (control, saline, 10 ml/kg/d), AHH group (model, saline, 10 ml/kg/d), Hongjingtian oral liquid group (HOL, 0.42 ml/kg/d, No. Guoyaozhunzi B20070002, Tiben Tibetan Medicine Group Co., Ltd), and three BRP groups (BRP, 38, 75, and 150 mg/kg/d, respectively). The rats in the BRP groups received respective doses of BRP, while the rats in the control and model group received saline. The rats were administered treatment by oral gavage for seven consecutive days. One hour after the last administration, all rats were subjected to AHH stimulation except for the control group. The plateau environmental conditions were mechanically simulated using an animal compound environment test chamber (FLYDWC50-II C; Avic Guizhou Fenglei Aviation Armament Co., Ltd., Anshun, China) after 30 min acclimatization (simulated environment: rising to 3000 m at a speed of 5 m/s for 30 min, subsequently rising to 4500 m for 30 min, then rising to 9000 m for 23 h, and finally fall to initial altitude at the same speed; gas flow rate: 0.9 L/min; temperature: 15-17°C; relative humidity: 55-60%).

After simulating AHH for 24 h, rats were sacrificed, followed by blood and brain samples were collected.

### 2.5. Serum Parameters Analysis

The blood samples were collected from the abdominal aorta. Serum samples were separated by centrifugation at 3500 rpm at 4°C for 10 min. The levels of SOD, MDA, T-GSH, GSSG, and LDH in serum were detected with the corresponding kits, and the concentration of GSH was calculated as GSH = T-GSH -2 × GSSG.

### 2.6. H&E Staining and Nissl Staining

Samples of the left hippocampus and cortex from rat brain were rinsed with precooled saline, fixed in 4% paraformaldehyde solution, dehydrated with different concentration gradient ethanol, then embedded with paraffin and sliced into sections using a microtome (RM2235, Leica Biosystems, Wetzlar, Germany), finally stained with hematoxylin and eosin and 0.1% toluidine blue, respectively. The pathological changes and the number of Nissl bodies in hippocampus and cortex were observed at 200× and 400× using a light microscope (BA400, Digital, Motic China Group Co., Ltd, Guangzhou, China).

### 2.7. TUNEL Staining

The paraffin-embedded sections were dewaxed, hydrated, and incubated for 10 min at room temperature with 3% H_2_O_2_ solution. Then, the sections were processed with proteinase K solution at 37°C for 15 min and washed in TBS for three times (5 min each time). Next, marking buffer (1 *μ*L TdT, 1 *μ*L DIG-d-UTP, 18 *μ*L labeling Buffer) was mixed with the sections and labeled at 37°C for 2 h, and washed in TBS for 3 times (2 min each time). The sections were added with blocking solution (50 *μ*L) for 30 min, followed by biotinylated anti-digoxin antibody (1 : 100, 50 *μ*L) for 30 min at 37°C. After washing with TBS for 3 times, the SABC (1 : 100, 50 *μ*L) was added in the sections for 30 min at 37°C, and rinsed with TBS for 4 times (5 min each time). Finally, DAB was used for color rendering for 10 min and washed with PBS for 3 times, and the sections were processed with counterstain, dehydration, transparency, and seal. Sections were photographed under a 200× microscope and TUNEL-positive nuclei were counted with Image-Pro Plus 6.0 software. The TUNEL index was determined by calculating the number of positive stained cells per mm^2^.

### 2.8. Western Blot Analysis

Western blot was used to detect the expression of p-PI3K, PI3K, p-Akt, Akt, HIF-1*α*, Bax, Bcl-2, Caspase-3, and *β*-actin in the nucleus of rat brain nerve cells. The right brain tissue of rats was rinsed with PBS, transferred to a 2 mL EP tube, and added lysates. After being cut into pieces with scissors, the brain tissue was cleaved in an ice bath for 10 min, centrifuged at 4°C for 10 min at 12,000 rpm, and the total protein concentration of the supernatant was detected using BCA protein concentration determination kit. A total of 60 *μ*g protein per well was separated using sodium dodecyl sulfate-polyacrylamide gel electrophoresis (SDS-PAGE, 10%) and transferred to polyvinylidene difluoride (PVDF) membranes. Then, PVDF membranes were blocked with 5% nonfat milk in TBST for 1 h and incubated overnight at 4°C with each primary antibody: mouse PI3K (1 : 1000), p-PI3K (1 : 1000), Akt (1 : 1000), p-Akt (1 : 500), HIF-1*α* (1 : 1000), Bax (1 : 1000), Bcl-2 (1 : 1000), Caspase-3 (1 : 500), and *β*-actin (1 : 10000). Membranes were washed 3 times with TBST for 5 min each and then incubated with horseradish peroxidase-conjugated goat anti-rabbit immunoglobulin G secondary antibody (1 : 1000) at 25°C for 2 h. After washing with TBST, membranes were revealed by ECL-Plus detection kit and images were captured using ChampChemi 610 Plus. Density values of the blots were analyzed using Image-Pro Plus software version 6.0 (Media Cybernetics, Inc.) and expressed as percentage of *β*-actin.

### 2.9. Quantitative Real-Time PCR (qRT-PCR) Analysis

TRIzol reagent was used to extract total RNA. The reverse transcription kit indicated the synthesis of cDNA. The qRT-PCR detection was performed using fluorescence quantitative PCR instrument (QTOWER2.2, Jena Analytical Instruments GMBH). PCR amplification was performed for 39 cycles as following: 3 min at 95°C for initial activation, 10 s at 95°C for denaturation, 10 s at 58°C for annealing/extension. The primers for microRNA-210, ISCU1/2, COX10, and Caspase-3 were designed using Beacon Designer version 7.92 and PRIMER 5 software, and the information of target gene is listed in Table 1. Gene expression was normalized to the expression of GAPDH and the relative gene expression was calculated using the 2^-∆∆Ct^ method.

### 2.10. UPLC-MS-Based Brain Metabolomics Studies

The brain tissue samples of the control, model, and BRP 150 mg groups were thawed at room temperature 2 h before experiment. Then, brain tissue samples (100 mg), 80% methanol (1000 *μ*L, stored at -20°C), and 8 ppm 2-chlorophenylalanine (60 *μ*L, internal standard substance) were added in an EP tube. After centrifuged at 14,000 rpm and 4°C for 10 minutes using tissue lapping apparatus, the supernatant (800 *μ*L) was placed in an EP tube and concentrated by a centrifugal concentrator. Next, the concentrated samples were dissolved with 50% methanol (400 *μ*L, stored at 4°C) and filtered, then the filtrate (20 *μ*L) of each sample to be tested was mixed for quality control (QC).

Chromatographic conditions: LC system (Thermo Ultimate 3000) combined with ACQUITY UPLC® HSS T3 C18 column (1.8 *μ*m, 2.1 × 150 mm, Waters) was used for sample separation. The mobile phases of positive ionization mode were water with 0.1% formic acid (A) and acetonitrile with 0.1% formic acid (B); the mobile phases of negative ionization mode were water with 5 mM ammonium formate (C) and acetonitrile (D). The flow rate was set at 0.25 mL/min, and the initial composition was 2% B/D; this was followed by a linear increase to 50% B/D, which was maintained for 8 min, and then increased to 98% B/D over 3 min; column temperature was set at 40°C; automatic sampler was set at 8°C, and loading volume was set at 2 *μ*L.

MS conditions: electrospray ionization (ESI); mass spectrometer (Thermo Q Exactive Focus) positive ionization mode (3.80 kV), negative ionization mode (2.50 kV); sheath gas, 45 arb; auxiliary gas, 15 arb; capillary temperature, 325°C; resolution, 70000; scanning range, 81-1000 amu; dissociation mode, HCD (30 eV collision energy).

### 2.11. Statistical Analysis

All statistical analysis was performed with SPSS 17.0 (Media Cybernetics, Inc., Rockville, MD, USA) software. Data were expressed as the mean value ± standard deviation. Statistically significant differences between groups were determined by one-way ANOVA analysis. *P* values <0.05 were considered statistically significant. All images were processed with GraphPad Prism 5.0 (GraphPad Software Inc., San Diego, CA, USA) software. Whereas, for the analysis of metabolites, the raw data from UPLC-Q-Exactive-MS analysis were converted to mzXML fomat using Proteowizard software (v3.0.8789), then further processed by R XCMS (v3.3.2) for peaks identification, filtration, and alignment. The data was imported into SIMCA-P 14.1 for principal component analysis (PCA), supervised partial least squares discriminant analysis (PLS-DA), and orthogonal partial least squares discriminant analysis (OPLS-DA). The different metabolites were selected based on VI*P* value of S-plot (VIP>1) and T-test (*P* <0.05). For the identification of potential markers and metabolic pathway, the following databases have been used: HMDB (http://www.hmdb.ca/), Metlin (http://metlin.scripps.edu), massbank (http://www.massbank.jp/), Metabo Analyst (http://www.metaboanalyst.ca/), mzclound (https://www.mzcloud.org), LipidMaps (http://www.lipidmaps.org), mzclound (https://www.mzcloud.org), KEGG database (http://www.genome.jp/kegg/), and references.

## 3. Results

### 3.1. Effects of BRP on Oxidative Stress Index in Serum

**Figure 1:**
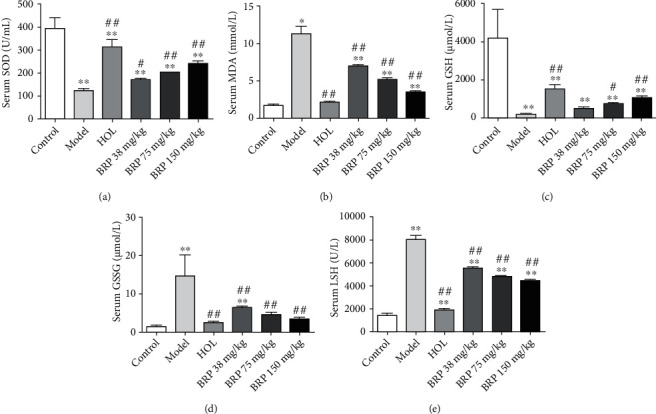
Effect of BRP administration on serum SOD (a), MDA (b), GSH (c), GSSG (d), and LDH (e) levels. Data are presented as the mean ± SD; *n* =6. Control and model groups were administered saline. ^∗^*P* < 0.05 or ^∗∗^*P* < 0.01 versus control group; ^#^*P* < 0.05 or ^##^*P* < 0.01 versus model group. SOD: superoxide dismutase; MDA: malondialdehyde; GSH: glutathione; GSSG: oxidized glutathione; LDH: lactate dehydrogenase.

To evaluate the antioxidant activity of BRP, the contents SOD, MDA, GSH, GSSG, and LDH in serum of rats were detected by ELISA kits. As shown in Figure 1, compared with the control group, the levels of SOD and GSH in the model group were significantly decreased, while the levels of MDA, GSSG, and LDH were remarkably increased. However, the treatment with HOL and BRP caused a marked increase in levels of SOD and GSH and a decrease in levels of MDA, GSSG, and LDH with a dose-dependent manner.

### 3.2. Improvement of BRP on Neuron Injury, and Cell Vitality after Hypoxic Insult

**Figure 2:**
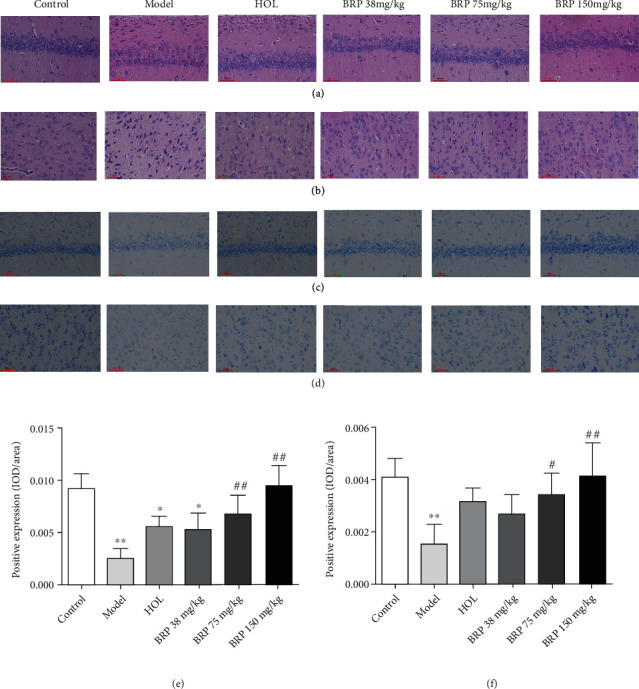
Effects of BRP on AHH-induced brain injury and neuronal viability in rats. (a) H&E-stained sections of the hippocampal CA1 region. (b) H&E-stained sections of the cortical region. (c) Nissl-stained neurons of the hippocampal CA1 region. (d) Nissl-stained neurons of the cortical region. (e) Quantitative analysis of Nissl staining in hippocampal CA1 region. (f) Quantitative analysis of Nissl staining in cortical region. Magnification: 200x. Scale bar: 50 *μ*m. Data are presented as the mean ± SD; *n* =6. ^∗^*P* < 0.05 or ^∗∗^*P* < 0.01 versus control group; ^#^*P* < 0.05 or ^##^*P* < 0.01 versus model group.

To determine the effect of BRP on hippocampal and cortical areas of rats in brain tissue, H&E staining and Nissl staining were used to observe the pathological changes of neuron cells and Nissl bodies. As shown in Figures 2(a) and 2(b), compared with control group, there were a large number of atrophic pyramidal cells of neurons, with evident intercellular spaces widening, nuclei in irregular shapes in the hippocampal CA1 area in the model group. Meanwhile, there was shrinkage of cortical neurons, deep staining of nuclei in the cortical area. However, HOL and BRP administration ameliorated these pathological changes.

The Nissl staining showed (Figures 2(c)–2(f)) abundant Nissl bodies with a normal, clear cytoplasm in neurons of the control group. However, the hippocampal CA1 and cortical neurons were damaged, the number of the Nissl bodies decreased, and the staining became blurred after hypoxic insult. Interestingly, BRP treatment alleviated these damages of neurons.

### 3.3. Effect of BRP on Apoptosis of Rat Nerve Cells

**Figure 3:**
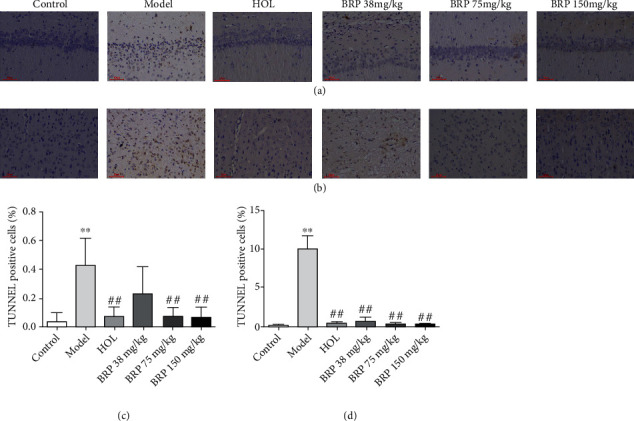
Effects of BRP on AHH-induced neuron apoptosis in rats. (a) TUNEL-positive apoptotic cell staining of hippocampal CA1 region. (b) TUNEL-positive apoptotic cell staining of cortical region. (c) Analytical result of TUNEL-positive cells in hippocampal CA1 region. (d) Analytical result of TUNEL-positive cells in cortical region. Magnification: 200x. Scale bar: 50 *μ*m. Data are presented as the mean ± SD; *n* =6. ^∗^*P* < 0.05 or ^∗∗^*P* < 0.01 versus control group; ^#^*P* < 0.05 or ^##^*P* < 0.01 versus model group.

To investigate the anti-apoptotic effect of BRP in AHH rats, TUNEL staining was used to examine cells apoptosis. As presented in Figure 3, the hippocampal CA1 and cortical neurons in the control group were orderly, with normal structure and clear nucleus. By contrast, a large number of apoptotic positive cells were observed in hippocampal CA1 and cortical area in the model group, with shrinking nuclei and concentrated cytoplasm. Compared to the model group, the morphology of nerve cells in BRP groups was normal and clear, and apoptotic positive cells decreased significantly.

**Figure 4:**
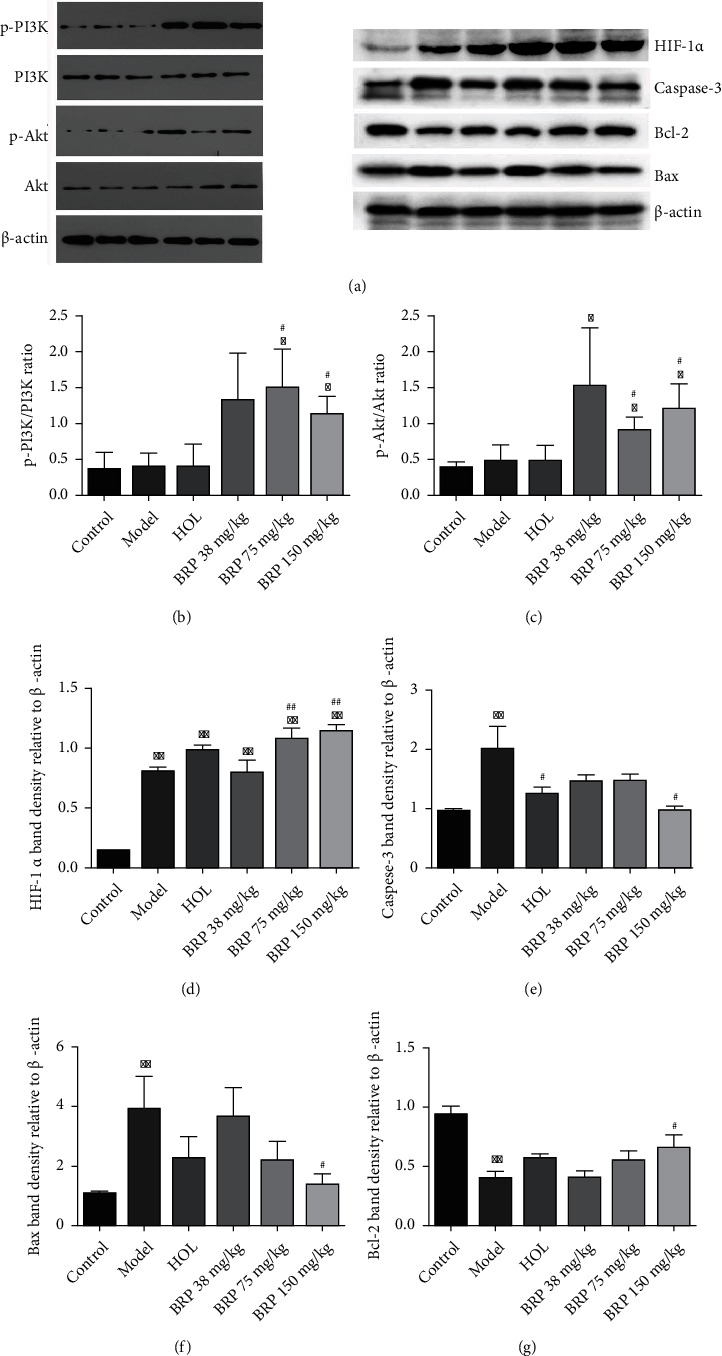
Effects of BRP on PI3K/Akt/HIF-1*α* signaling pathway by using western blotting. (a) Relevant protein bands. (b) p-PI3K/PI3K. (c) p-Akt/Akt. (d) HIF-1*α*. (e) Caspase-3. (f) Bcl-2. (g) Bax. Data were normalized against *β*-actin and expressed as a relative value. Data are presented as the mean ± SD. ^∗^*P* < 0.05 or ^∗∗^*P* < 0.01 versus control group; ^#^*P* < 0.05 or ^##^*P* < 0.01 versus model group. PI3K: phosphatidylinositol-3-kinase; Akt: protein kinase B; HIF-1*α*: hypoxia-inducible factor; Caspase-3: cysteine-aspartic proteases-3; Bcl-2: B-cell lymphoma-2; Bax: Bcl-2 associated X protein.

**Figure 5:**
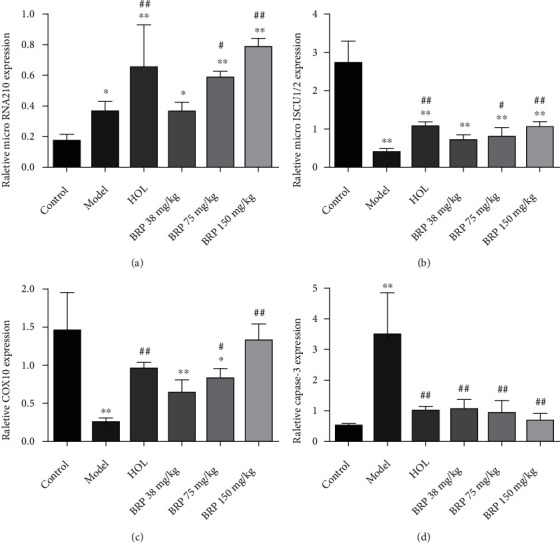
Effect of BRP on the expression of microRNA210 (a) and mRNA levels of ISCU1/2 (b), COX10 (c), Caspase-3 (d) in cerebral tissue of rats. Data were normalized against GAPDH and expressed as a relative value. Data are presented as the mean ± SD, *n* =6. ^∗^*P* < 0.05 or^∗∗^*P* < 0.01 versus control group; ^#^*P* < 0.05 or ^##^*P* < 0.01 versus model group. ISCU1/2: iron-sulfur cluster scaffold; COX10: cytochrome c oxidase assembly protein; Caspase-3: cysteine-aspartic proteases-3.

Subsequently, the expression levels of apoptosis-related proteins Bax, Bcl-2, and Caspase-3 in different groups were detected by western blotting. As shown in Figures 4(e)–4(g), the hypoxia upregulated Caspase-3 and Bax protein levels, with an obvious reduction after BRP administration. Moreover, the expression of Bcl-2 protein decreased after hypoxia while BRP treatment enhanced its expression. In addition, qRT-PCR also detected that BRP decreased the gene expression of Caspase-3 (Figure 5(d)).

### 3.4. BRP Regulated the PI3K/Akt/HIF-1*α* Signaling Pathway

Western blotting and qRT-PCR analysis were performed to examine the effect of BRP treatment on PI3K/Akt/HIF-1*α* signaling pathway. As presented in Figure 4, western blotting results revealed that BRP treatment increased phosphorylation of PI3K and Akt, and further upregulated the expression of HIF-1*α*. Moreover, overexpression of microRNA-210, COX10, and ISCU1/2 gene levels was observed after BRP administration compared with the model group by qRT-PCR analysis (Figures 5(a)–5(c)). These evidences suggest that BRP activates PI3K/Akt/HIF-1*α* signaling pathway to protect AHH-induced brain injury.

### 3.5. Multivariate Data Analysis

**Figure 6:**
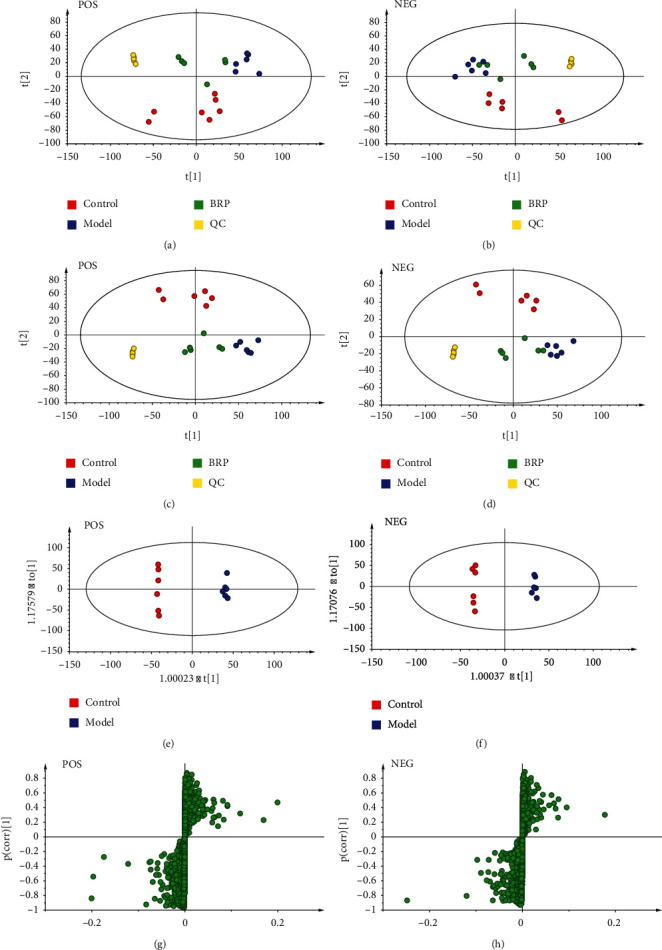
Multivariate data analysis from UPLC-Q-Exactive-MS. (a) Positive mode of PCA scores plot: R2X =0.536, Q2 = 0.228. (b) Negative mode of PCA scores plot: R2X =0.510, Q2 = 0.307. (c) Positive mode of PLS-DA scores plot: R2X =0.600, R2Y =0.992, Q2 = 0.795. (d) Negative mode of PLS-DA scores plot: R2X =0.637, R2Y =0.991, Q2 = 0.850. (e) Positive mode of OPLS-DA score plots: R2X =0.824, R2Y =1, Q2 = 0.682. (f) Negative mode of OPLS-DA score plots: R2X =0.868, R2Y =0.999, Q2 = 0.575. (g) S-plots of the control and model groups in positive mode. (h) S-plots of the control and model groups in negative mode.

Brain metabolic profiling in the control group, model group, and BRP group was assessed by multivariate analysis. PCA was used to investigate whether the three groups could be separated and to reveal their metabolic differences (Figures 6(a) and 6(b)). The PCA score scatter plots showed that the control group clearly separated from the model group and BRP group, but the separation trend between model group and BRP group was not obvious. The reason for this may be that environmental factors lead to there being no differences between groups. In order to exclude the metabolic changes caused by some factors unrelated to the experiment and obtain more accurate results, the supervised PLS-DA was used as a pattern recognition method to observe the distribution and visualize metabolic differences among the three groups. As shown in Figures 6(c) and 6(d), the PLS-DA score plots indicated that the control, model, and BRP groups were clearly distinguished. The brain metabolites of the model were significantly different from those of the control group. After BRP treatment, BRP group tended to separate from the model group and move closer to the control group, which implied that the treatment effect of BRP was satisfactory. In addition, it can be seen in the PCA and PLS-DA score plots that QC samples are clustered, indicating that the system has high repeatability and good stability.

Then, OPLS-DA was applied to discriminate the differential metabolites contributing to the separation of the model group and the control group. As shown in Figures 6(e) and 6(f), it can be seen that the two groups can be clearly separated in positive and negative ion modes, indicating that metabolic abnormalities occurred in the AHH rats.

### 3.6. Identification of Metabolites and Pathway Analysis

The VI*P* value of S-plots (Figures 6(g) and 6(h)) and Student's t-test of the *P*-value can reflect the importance of the metabolites. Variables with VIP >1 and *P* <0.05 were considered the important selection criteria for identifying the potential differential biomarkers. The metabolites obtained from UPLC-Q-Exactive-MS analysis were identified according to MS/MS fragments, retention behavior, and online databases. Here in Table 2, a total of 15 potential biomarkers were determined. Compared with the control group, the levels of pyruvate, dropropizine, dicyclomine, and tetradecylamine were increased, and the levels of choline, flavin adenine dinucleotide, 2-sec-butyl-4,6-dinitrophenol, psychosine, sphingosine-1-phosphate, sphingosine, myristoylcarnitine, LysoPE (16 : 1(9Z)/0 : 0), LysoPA(16 : 0/0 : 0), LysoPA(18 : 1(9Z)/0 : 0), and 1-stearoyl-lysophosphatidylserine were decreased in the model group. A total of 7 metabolites including pyruvate, psychosine, sphingosine-1-phosphate, sphingosine, myristoylcarnitine, LysoPE (16 : 1(9Z)/0 : 0), and LysoPA(16 : 0/0 : 0) were intervened by BRP treatment.

**Figure 7:**
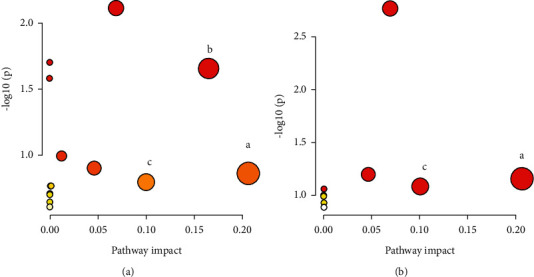
Metabolic pathways involved in potential markers in the brain. (A) Metabolic pathways affected by AHH. (B) Metabolic pathways of BRP. The pathway impact value greater than 0.1 was selected: (a) pyruvate metabolism; (b) glycerophospholipid metabolism; (c) glycolysis and gluconeogenesis.

The metabolic pathway was established by importing the potential metabolites into the Metaboanalyst database. The pathways with impact values above 0.1 were screened as potential target pathways. As shown in Figure 7, the pyruvate metabolism (impact 0.207), glycerophospholipid metabolism (impact 0.166), and glycolysis/gluconeogenesis (impact 0.100) are the most important metabolic pathways associated with AHH, and the correlation pathways of potential biomarkers in response to therapeutic effects of BRP on AHH were linked by pyruvate metabolism and glycolysis/gluconeogenesis.

## 4. Discussion

Hypobaric hypoxia can cause severe brain damage and mitochondrial dysfunction, and is involved in hypoxic brain injury [30]. The hippocampus and cortex are essential areas in the brain for learning and memory [22, 31]. It was reported that the hippocampus and cortex were seriously damaged, and the number of pyramidal cells was decreased in rat under AHH [32]. In this study, H&E staining was employed to determine the protective effect of BRP on hypoxia-induced neuronal injury. H&E staining revealed obvious histopathological changes after AHH compared to the control group. These histopathological changes included decreased number of neurons and widened cell space in the hippocampus and cerebral cortex. However, these defects were apparently alleviated by BRP treatment. Furthermore, to assess neuronal activity, the number and morphology of Nissl bodies were examined by Nissl staining. The results showed that the cellular morphology was relatively complete, and the number of Nissl bodies increased significantly after preventive administration of BRP compared to the model group. Taken together, these findings suggest that BRP can reduce AHH-induced neuronal damage and increase cell viability.

Hypoxia can induce an imbalance between free radical generation and antioxidant protection, resulting in oxidative damage to biomolecules. The previous study showed that after chronic intermittent hypoxia exposure, oxidative stress in substantia nigra, cortex, and hippocampus increased [33]. Meanwhile, it is reported that LDH, SOD, GSH, and MDA are related to cell injury, and SOD and MDA are also biomarkers of oxidative stress [34]. Additionally, under hypobaric hypoxia, the antioxidant defense system such as GSH, SOD, and GSH/GSSG levels was significantly decreased, while the MDA level significantly increased [35, 36]. In this study, preventive administration of BRP can increase the levels of SOD and GSH and reduce the levels of MDA, GSSH, and LDH in a dose-dependent manner. Our findings were in accordance with expected results, which suggest that BRP can effectively improve oxidative stress damage and restore the antioxidant function.

Many studies demonstrated that hypoxia could increase cell apoptosis [37–39]. Caspase-3 and Bcl-2 family proteins play an irreplaceable role in apoptosis [37]. The Bcl-2 family includes anti-apoptotic (Bcl-2) and proapoptotic (Bax) proteins, and the proportion of Bcl-2/Bax in cells determines sensitivity to cell apoptosis [30]. It is reported that hypoxia will increase the levels of Caspase-3 and induce apoptosis of pyramidal neurons in the hippocampal CA1 [40]. In addition, exposure to hypoxia increased Bax expression and decreased Bcl-2 expression [41]. To determine the inhibitory effect of BRP treatment on AHH-induced apoptosis, the expression of Bax, Bcl-2, and Caspase-3 proteins was therefore determined by western blotting. The results showed that BRP treatment decreased the expression of Caspase-3 and Bax proteins and increased the expression of Bcl-2. At the same time, the qRT-PCR result showed that high expression of Caspase-3 induces apoptosis, but BRP decreased the expression of Caspase-3. In addition, TUNEL staining showed that the morphology of nerve cells was normal and clear, and the apoptotic bodies were reduced after BRP treatment. These findings indicate that the protective effect of BRP on AHH-induced brain injury is related to its anti-apoptotic activity.

The PI3K/Akt signaling pathway is widely distributed in cells and plays a key role in the regulation of oxidative stress and apoptosis induced by hypoxia [42, 43]. When the body is in a hypoxic environment, the expression of HIF-1*α* increases significantly in the nervous system [44]. Previous studies have shown that HIF-1*α* is subjected to regulation by the PI3K/Akt [13–15]. Activation of HIF-1*α* is an important signal of the hypoxia response in tissues [45]. Furthermore, mitochondria also play an important role in hypoxia induced apoptosis and oxidative stress [46, 47]. MicroRNA-210 is regulated by HIF-1*α* under hypoxic conditions and controls mitochondrial energy metabolism by repressing the iron-sulfur cluster assembly protein ISCU1/2 [48]. COX10, another factor of the mitochondria electron transport, has been identified as potential targets of microRNA210 [49]. It has been reported that activation of PI3K/Akt could alleviate hypoxic injury and further upregulate HIF-1*α* at the protein level [15, 50–52]. By contrast, hypoxia induced HIF-1*α* accumulation could stimulate the excessive production of microRNA-210 and inhibit mitochondrial respiration, which is accompanied by the downregulation of ISCU1/2 and COX10 expression [49, 53]. Our study revealed increases in HIF-1*α* and miR-210 expression, and decreases in ISCU1/2 and COX10 expression after hypobaric hypoxia stimulation. Interestingly, BRP administration stimulated p-PI3K/PI3K, p-Akt/Akt, HIF-1*α*, and microRNA-210 overexpression, while increasing gene levels of ISCU1/2 and COX10 in rats. Our research findings were consistent with previous research, illustrating that PI3K/Akt/HIF-1*α* activation could reduce oxidative stress, inhibit apoptosis, and alleviate brain injury. However, whether the increase of HIF-1*α* protein expression induced by BRP is due to the activation of protein synthesis pathway or the inhibition of protein degradation pathway remains to be determined.

Metabonomics technology provides a good method for the study of the mechanism of high-altitude hypoxia, overcomes the shortcomings of single component and single target in the traditional study of hypoxia mechanism, and can verify the effectiveness of drugs from the metabolic pathway. The previous study revealed that hypobaric hypoxia caused significant and comprehensive metabolic changes, and several key metabolic pathways such as pyruvate metabolism, glycerophospholipid metabolism, sphingolipid metabolism, and glucose metabolism were seriously disturbed [54, 55]. In this paper, UPLC-Q-Exactive-MS and multivariate analysis were used to identify metabolic pathways affected by BRP. Metabolomics results revealed that the levels of key metabolites in the pyruvate metabolism, glycolysis/gluconeogenesis, and glycerophospholipid metabolism such as pyruvate, choline, LysoPE(16 : 1(9Z)/0 : 0), LysoPA(18 : 1(9Z)/0 : 0), and LysoPA(16 : 0/0 : 0) were perturbed by the hypobaric hypoxia environment. Glucose metabolism and pyruvate metabolism are the main ways for brain cells to produce energy [56]. And glycerophospholipid metabolism contributes to hypoxia adaptation [57]. Our study found that BRP can drive metabolites (pyruvate, psychosine, sphingosine-1-phosphate, sphingosine, myristoylcarnitine, LysoPE (16 : 1(9Z)/0 : 0), and LysoPA (16 : 0/0 : 0)) to normal levels by regulating pyruvate metabolism, glycolysis/gluconeogenesis, and so on. Interestingly, previous studies have shown that PI3K/Akt/HIF-1*α* signaling pathway is also involved in glucose metabolism [58]. This suggests that BRP may reverse the metabolic pathway through PI3K/Akt/HIF-1*α*. However, how BRP regulates pyruvate metabolism and glucose metabolism to achieve brain protection needs to be further studied.

## 5. Conclusion

Overall, BRP reduced hippocampal and cortical neuronic damage; ensured hippocampal and cortical neuronic viability; improved GSH and SOD activity while reducing expression levels of MDA, GSSG, and LDH; and preserved a high Bcl-2 expression while decreasing the level of Bax and Caspase-3. BRP also kept high expression of p-PI3K, p-Akt, HIF-1*α*, microRAN210, ISCU1/2, and COX10; and regulated pyruvate metabolism and glycolysis/gluconeogenesis. As a result, BRP has a clear protective effect on AHH-induced brain injury. Its mechanisms may be related to ameliorate oxidative stress injury and inhibit apoptosis via activating PI3K/Akt/HIF-1*α* signaling pathway and reverse metabolic pathway disturbances. The results on pharmacodynamics and metabolomics in this study could provide atheoretical basis for clarifying the mechanism of BRP in the treatment of AHH-induced brain injury.

## Figures and Tables

**Table 1:** The primers for the qRT-PCR.

Gene	Primer	Sequence
GAPDH	Primer (forward)	F: 5′-AGGTCGGTGTGAACGGATTTG-3′
	Primer (reverse)	R: 5′-GGGGTCGTTGATGGCAACA-3′
microRNA-210	Primer (forward)	R:5′-GTCGTATCCAGTGCAGGGTCCGAGGTATTC GCACTGGATACGACCAGTGT-3′
	Primer (reverse)	F:5′-GCCACGCCCACAGACAC-3′
COX10	Primer (forward)	R:5′- CAGGCTACAGAAGAACAG -3′
	Primer (reverse)	F:5′- CATCTCCCTCCCTATCAA -3′
ISCU1/2	Primer (forward)	R:5′- TAAGGAGCTGGAGGCAAT -3′
	Primer (reverse)	F:5′- GGTGGATGAAAAGGGGAAG -3′
Caspase-3	Primer (forward)	R:5′-TAGAGTAAGCATACAGGAAGT-3′
	Primer (reverse)	F:5′-TATTGAGACAGACAGTGGAA-3′

**Table 2:** The identification results of differential metabolite markers.

No.	T_R_/min	m/z	Chemical formular	Biomarkers	VIP	MG vs CG	TG vs MG	HMDB	Mode
1	1.43	87.01	C_3_H_4_O_3_	Pyruvate	1.710	↑∗	↓^##^	HMDB0000243	[M+H]-
2	1.5	104.11	C_5_H_14_NO	Choline	1.495	↓∗	—	HMDB0000097	[M+H]+
3	6.13	784.14	C_27_H_33_N_9_O_15_P_2_	Flavin adenine dinucleotide	2.019	↓∗∗	—	HMDB0001248	[M+H]-
4	8.41	237.16	C_13_H_20_N_2_O_2_	Dropropizine	1.912	↑∗∗∗	—	HMDB0251622	[M+H]+
5	11.8	239.07	C_10_H_12_N_2_O_5_	2-sec-butyl-4,6-dinitrophenol	1.608	↓∗	—	HMDB0032559	[M+H]-
6	12.85	462.34	C_24_H_47_NO_7_	Psychosine	1.630	↓∗	↑^#^	HMDB0000648	[M+H]+
7	12.88	310.27	C_19_H_35_NO_2_	Dicyclomine	1.898	↑∗∗	—	HMDB0014942	[M+H]+
8	13.07	214.25	C_14_H_31_N	Tetradecylamine	2.131	↑∗∗∗	—	HMDB0258887	[M+H]+
9	13.23	378.24	C_18_H_38_NO_5_P	Sphingosine-1-phosphate	1.748	↓∗	↑^###^	HMDB0000277	[M+H]-
10	13.45	300.29	C_18_H_37_NO_2_	Sphingosine	1.547	↓∗	↑^##^	HMDB0000252	[M+H]+
11	13.51	372.31	C_21_H_41_NO_4_	Myristoylcarnitine	1.468	↓∗	↑^#^	HMDB0254979	[M+H]+
12	13.54	452.28	C_21_H_42_NO_7_P	LysoPE (16:1(9Z)/0 : 0)	1.754	↓∗∗	↑^##^	HMDB0011504	[M+H]+
13	13.65	409.24	C_19_H_39_O_7_P	LysoPA (16 : 0/0 : 0)	1.609	↓∗	↑^#^	HMDB0007853	[M+H]-
14	13.8	435.25	C_21_H_41_O_7_P	LysoPA (18 : 1(9Z)/0 : 0)	1.675	↓∗	—	HMDB0007855	[M+H]-
15	13.89	524.3	C_24_H_48_NO_9_P	1-Stearoyl-Lysophosphatidylserine	1.828	↓∗	—	HMDB0240606	[M+H]-

*Note:* “↓” or “↑” represents the relatively decreased or increased levels of potential metabolite markers. MG vs CG represents the model group compared with the control group, ∗*P* < 0.05, ∗∗*P* < 0.01, ∗∗∗*P* < 0.001; and TG vs MG represents the BRP-treated group compared with the model group, ^#^*P* < 0.05, ^##^*P* < 0.01, ^###^*P* < 0.001.

## Data Availability

All data supporting the conclusions of this manuscript are provided in the text and figures. The datasets used and analysed during the current study are available from the author.

## Supplementary Materials

Supplementary Materials Supplementary 1: Supplementary Figure 4: uncropped photos of western blot.
