# Supplementary figures and images for: Protective Effect of Brassica rapa Polysaccharide against Acute High-Altitude Hypoxia-Induced Brain Injury and Its Metabolomics
Source: Oxid Med Cell Longev. 2022 Apr 23;2022:3063899. doi: 10.1155/2022/3063899 (PMC11401678; doi:10.1155/2022/3063899)

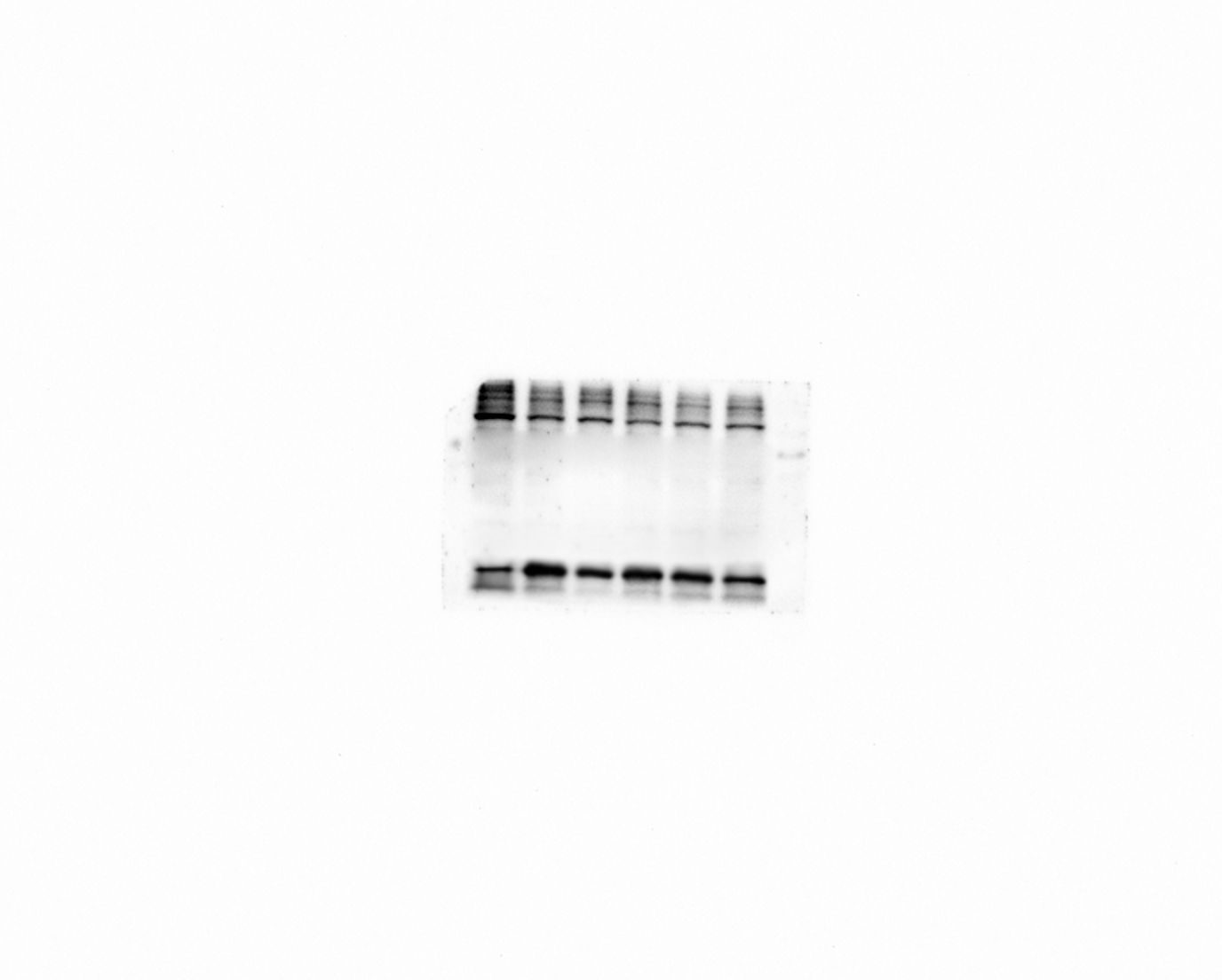


**Caspase-3**


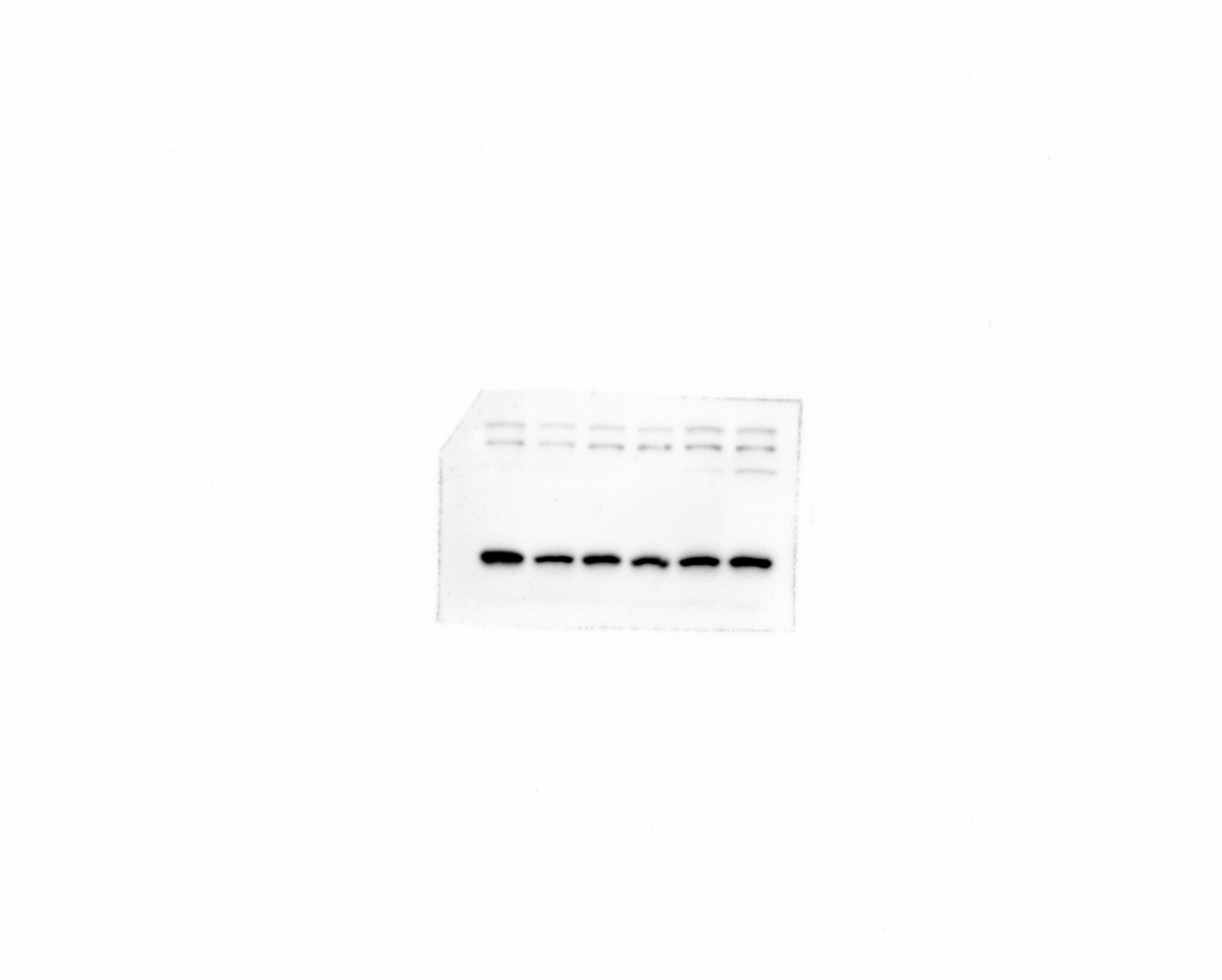


**Bcl-2**


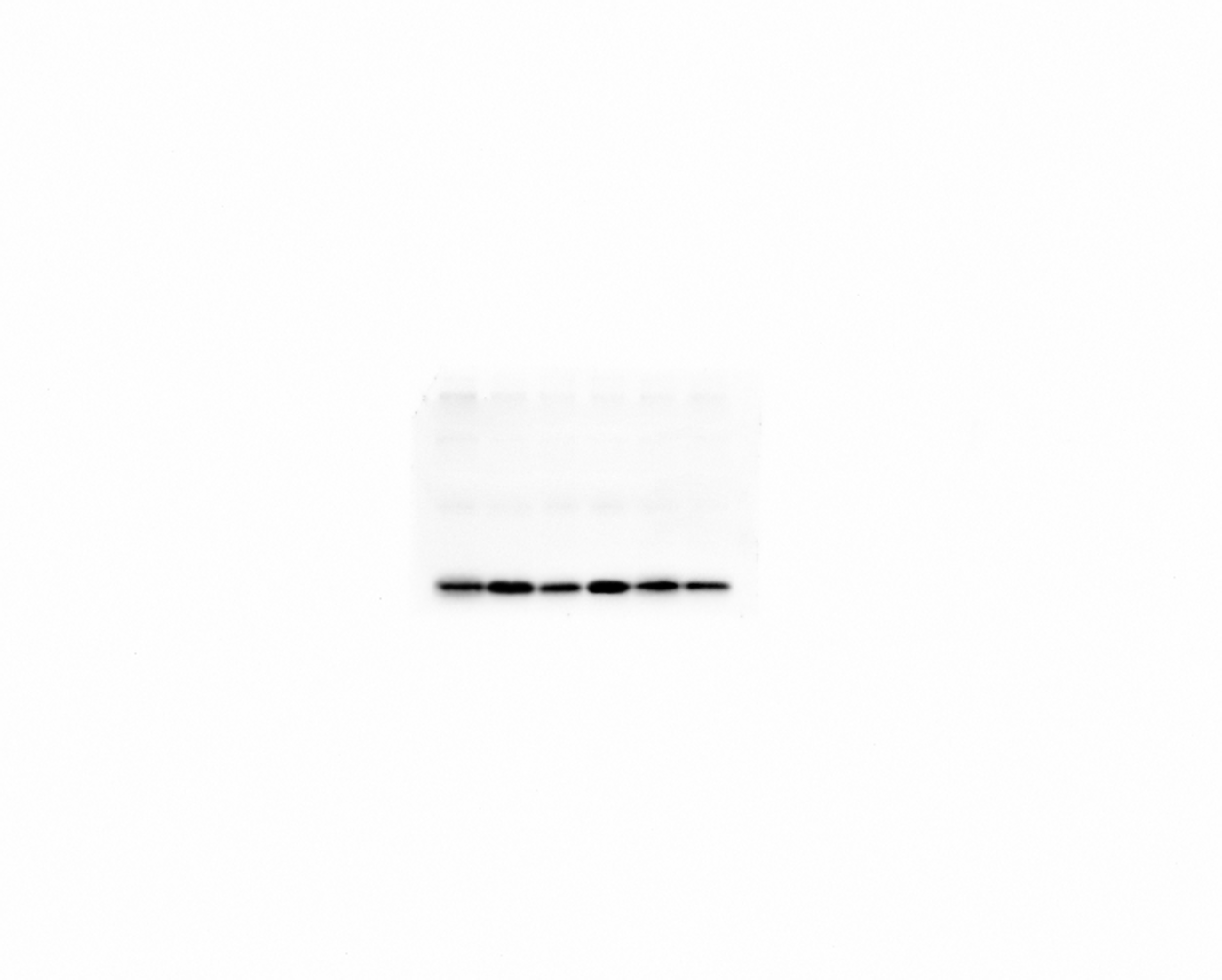


**Bax**

**
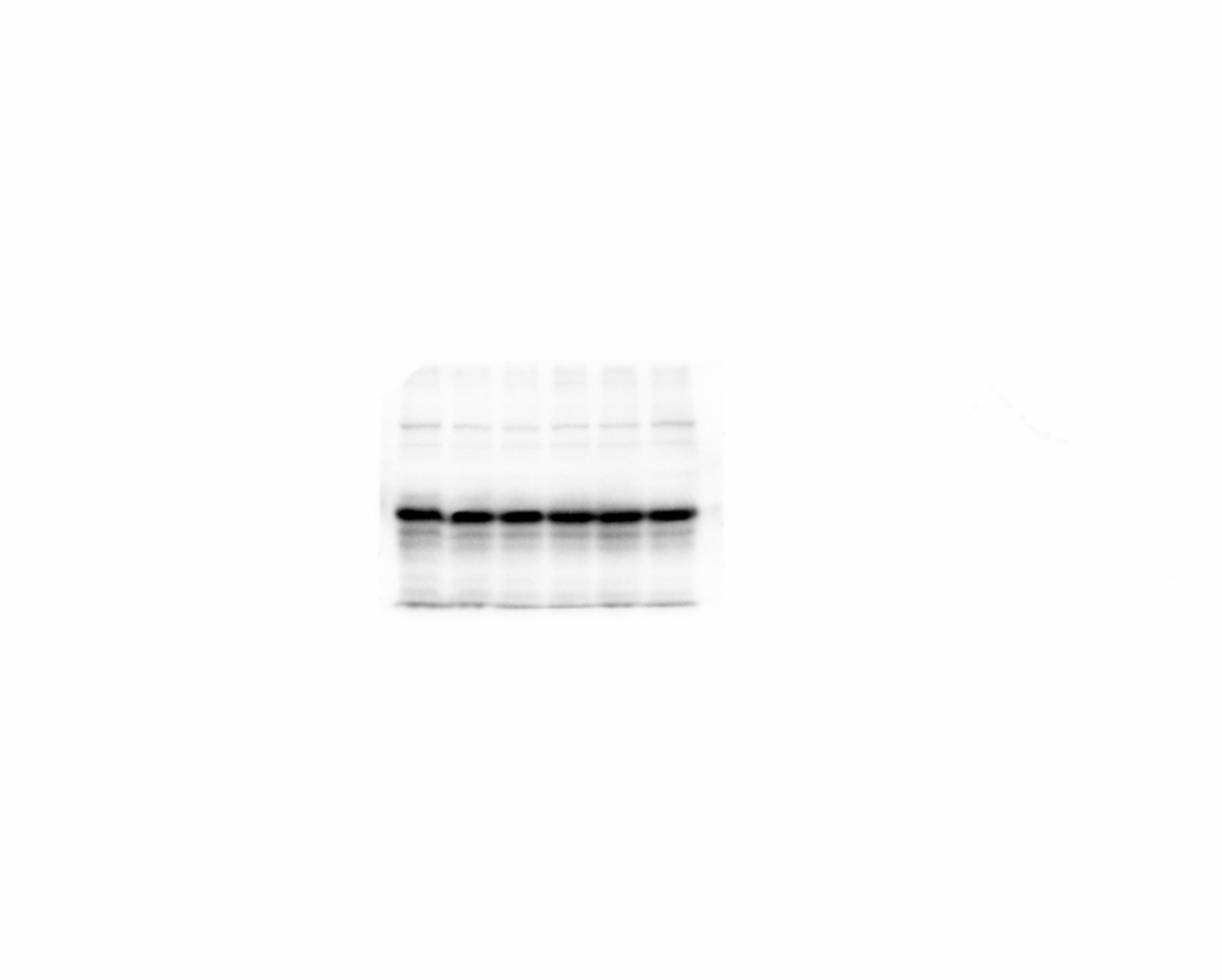
**

**β-actin**


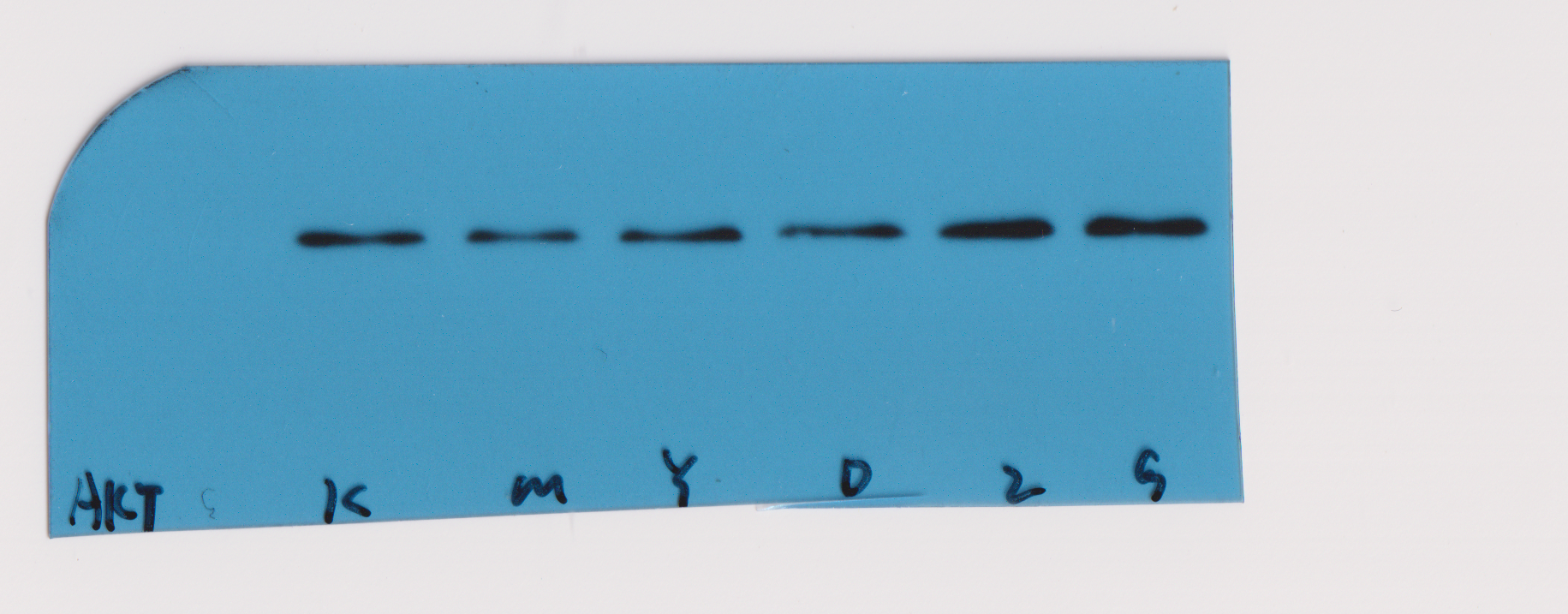


**Akt**


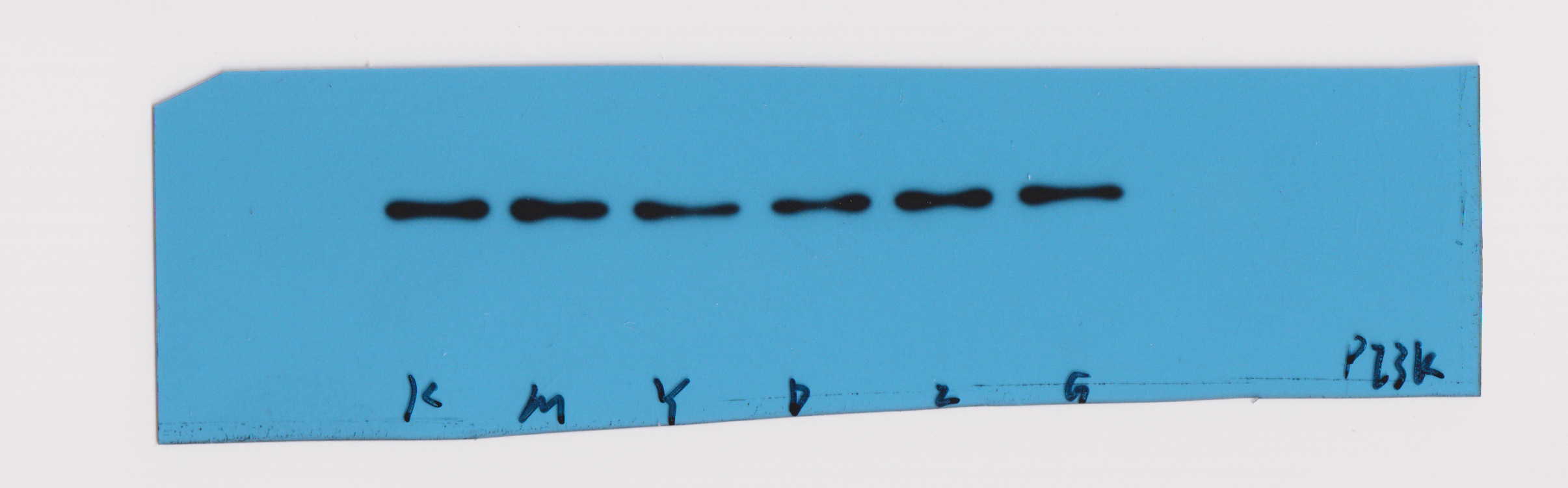


**PI3K**


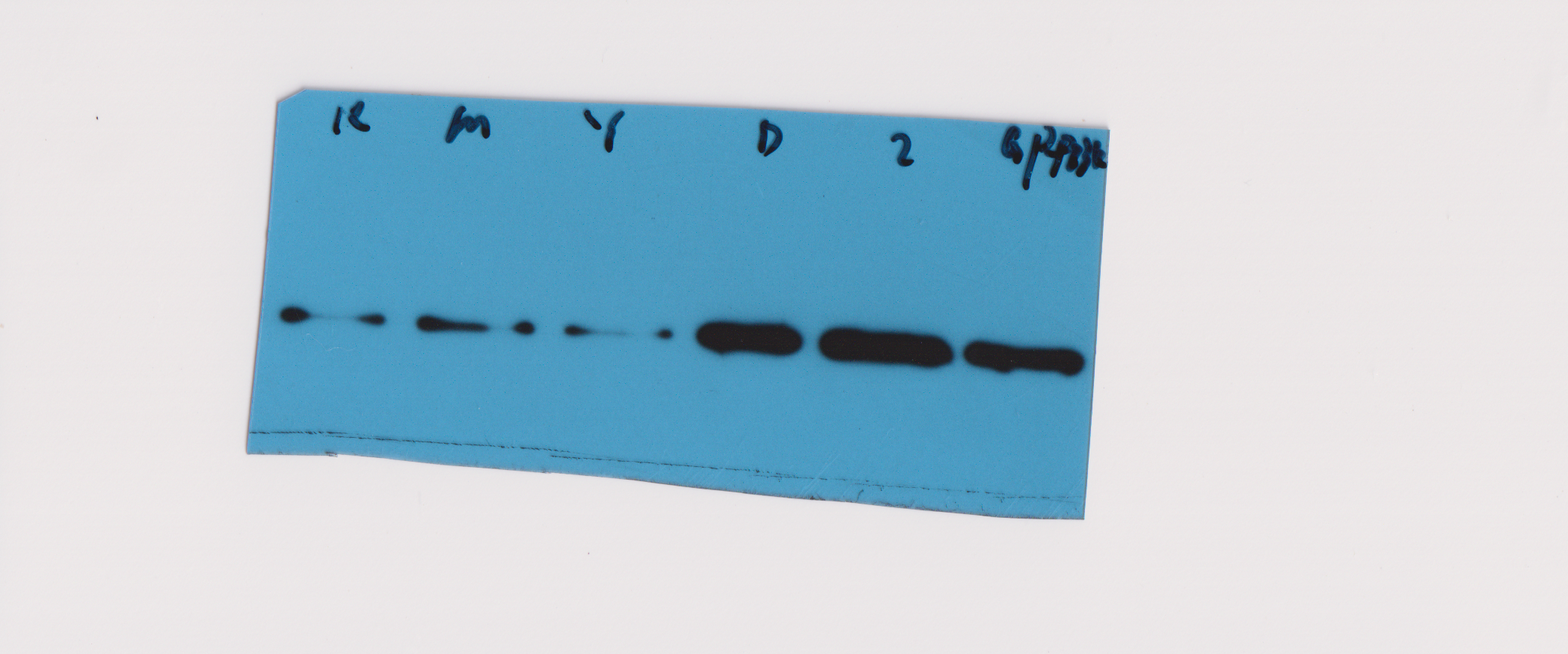


**p-PI3K**


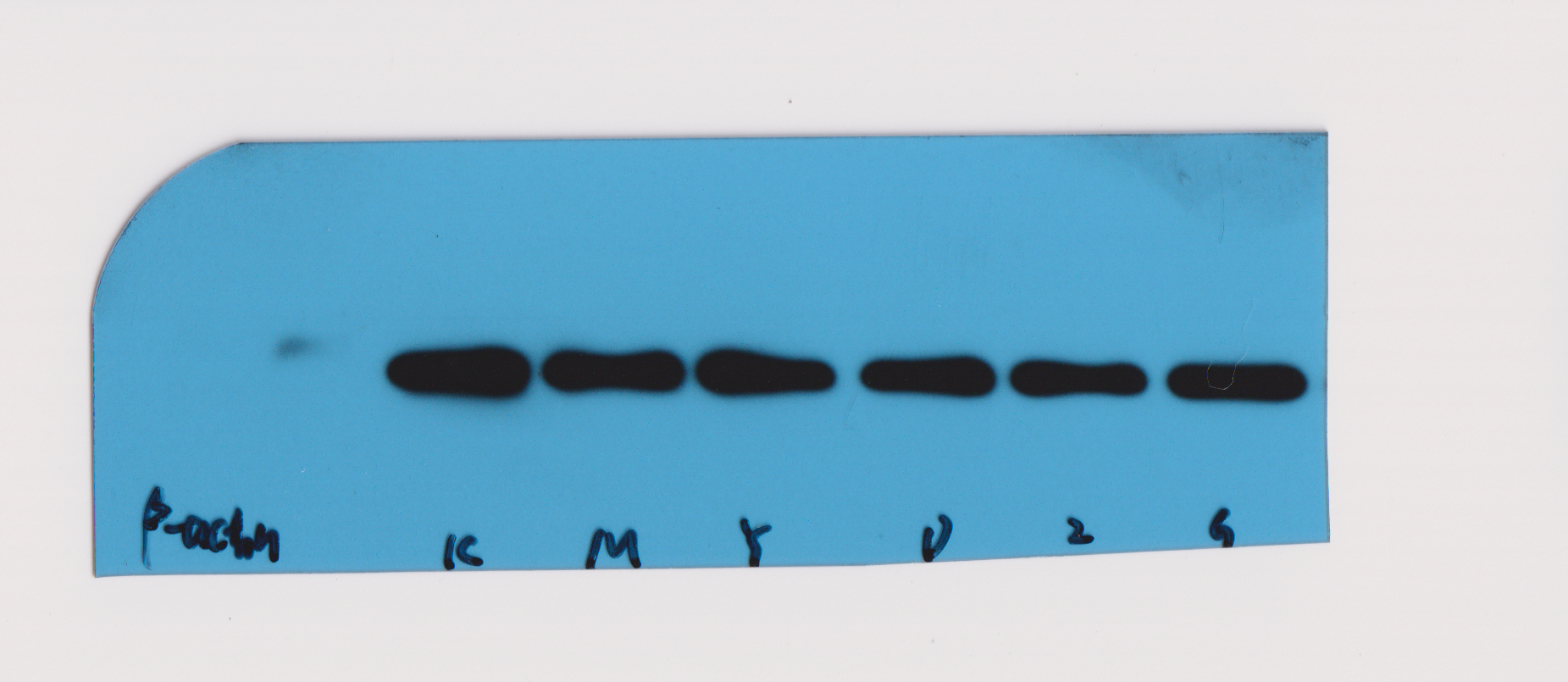


**β-actin**


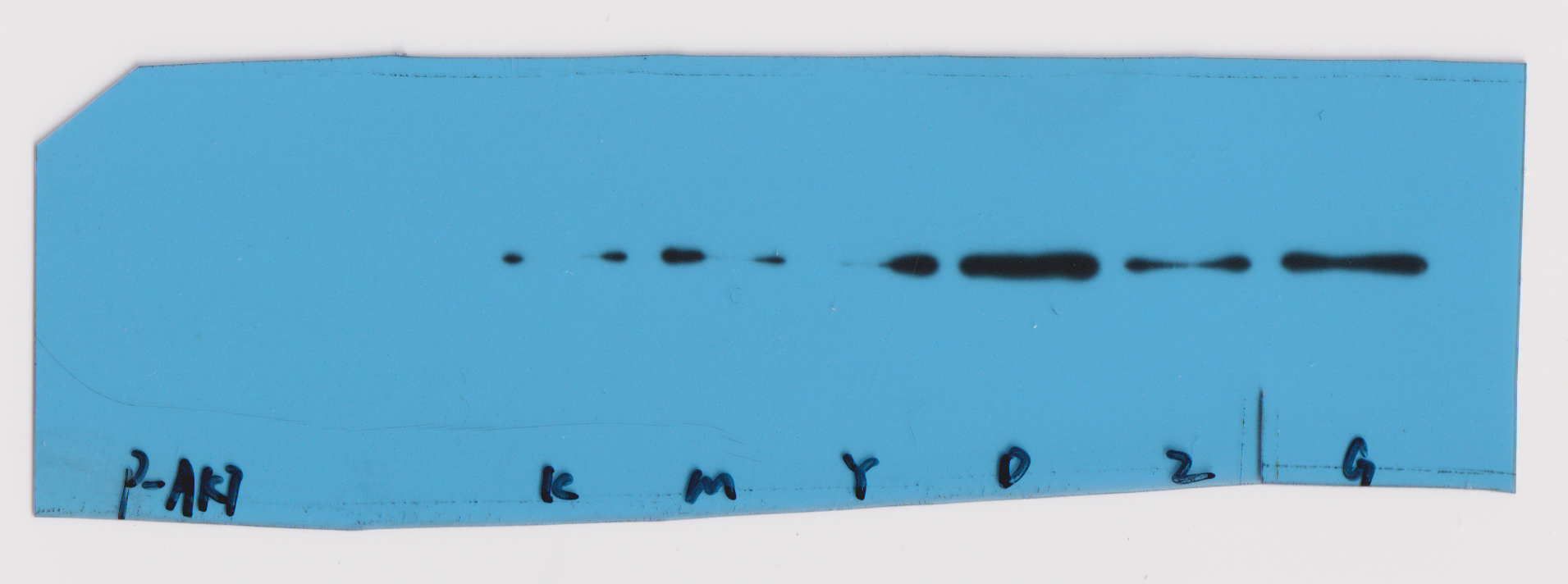


**p-Akt**

Supplement: Supplementary Materials — Supplementary 1: Supplementary Figure 4: uncropped photos of western blot. [file 3063899.f1.docx]
